# A Mild Alkaline Hydrothermal Etchant for MBene Exfoliation

**DOI:** 10.1021/acs.inorgchem.5c01969

**Published:** 2025-09-11

**Authors:** Yiren Zhang, Abraham A. Rosenberg, Joseph T. Doane, William Rice, Alma Kolakji, Michael T. Yeung

**Affiliations:** Department of Chemistry, 1084 University at Albany SUNY; Albany, Albany, New York 12222, United States

## Abstract

MoBene (the boride equivalent of MXenes) has been a synthetic challenge because borides degrade in common etching media. Inspired by the intercalation of lithium ions into AlOOH, we hypothesized that a hydrothermal route enables lithium ions to intercalate the aluminum layer of MoAlB and further separate the MoB sheet apart. Here, MoBene is first synthesized through a mild hydrothermal etching method with dilute lithium hydroxide. X-ray diffraction showed a peak at 8.9°, confirming an exfoliated layered structure, and the presence of MoBene sheets is further confirmed by SEM. Intriguingly, MoBene sheets, as the crystallographic subunit of the parent monoboride structure, can be readily restacked to crystalline MoB by heating in a mildly reducing atmosphere, which further confirmed the crystallinity of our product and demonstrated the ready synthesis of refractory materials at low temperature. The exfoliated MoBene product has chelating hydroxyl functional groups and can adsorb 318 mg/g of lead in water.

## Graphical abstract

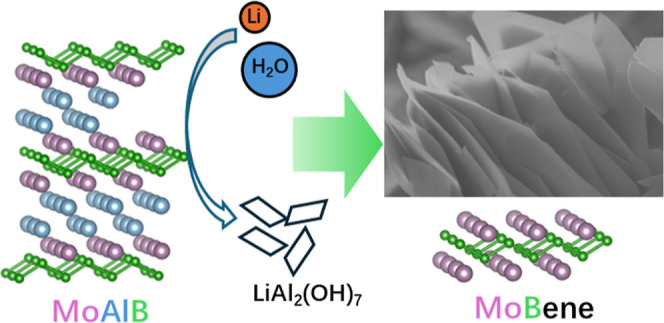

## 1 Introduction

MXenes are a class of layered materials that have found broad applications ranging from transparent conductors, electromagnetic interference shielding, humidity sensors, heavy metal adsorbents, and batteries.^1–6^ These layered materials are unique compared to traditional van der Waals materials, such as boron nitride or molybdenum disulfide, in that they are metallic in nature and topotactically derived from their crystallographically related parents. They are prepared from etching MAX phases, which possess a general formula of M_*n*+1_AX_*n*_, where M is an early transition metal, A is usually aluminum, and X is usually carbon (and in some rare cases nitrogen). MAX phases are etched to MXenes by stoichiometry M_*n*+1_X_*n*_T_*x*_, where *n* = 1, 2, or 3 and T is a terminal functional group such as –O, –OH, or –F. Unfortunately, despite aluminum being one of the easiest elements to chemically etch (it is amphoteric and is susceptible to both basic and acidic attack), the removal of electropositive elements from a MAX phase is challenging and requires exceptionally harsh (and in most cases toxic) conditions.

2-D Ti_3_C_2_T_2_ was first synthesized by selective etching with concentrated hydrofluoric acid (HF) from the Ti_3_AlC_2_ phase in 2011.^7^ Since then, many MAX phases were successfully etched with HF, such as V_2_CT_2_, Nb_2_CT_2_, and Ta_4_C_3_T_2_.^8–11^ However, HF and other fluorine-related etching methods are corrosive and toxic, which has inspired modestly safer approaches. For example, Ti_3_C_2_T_2_ sheets are successfully obtained by etching with HN_4_HF_2_^1^ and LiF/HCl^12^ solutions, but they generated HF in situ, and thus, these manuscripts often contain a safety warning to this effect. Fluorine was completely avoided when Ti_3_AlC_2_ was etched with a caustic 27.5 M NaOH solution heated to 270 °C to obtain a multilayered Ti_3_C_2_T_*x*_.^13^ A concentrated KOH paste can also produce MXene Ti_3_C_2_T_*x*_.^14^ Although the alkali method is successful, the high concentration of caustic base is hazardous and can lead to the degradation of MXene sheets. Alternatively, MXene can be synthesized with an energy-intensive molten salt method: Liu et al.^15^ explored how molten CuCl_2_ at 680 °C can be used as a Lewis acid etchant. These etching methods that have been applied to MXene synthesis thus far rely on rather extreme conditions, such as toxicity, corrosiveness, and high temperatures. Moreover, MXenes have been compositionally limited primarily to carbides; nitrides are difficult to exfoliate and borides are essentially nonexistent.

MBene is a new category of 2-D transition metal boride that is structurally similar to MXene:^16–18^ it can be obtained by exfoliation of the corresponding MAB phase material.^19^ To date, there are several papers reporting that MBenes have extraordinary properties, such as strong mechanical performance,^20^ electrochemical energy storage,^21^ and electrocatalysis.^8^ Unfortunately, all of the aforementioned applications are solely theoretical, as MBenes are difficult to synthesize. MoBene was prepared via molten salt, but the resulting sheets were heavily oxidized owing to the harsh high-temperature processing conditions.^22^ Likewise, scandium-doping in (Mo_2/3_Sc_1/3_)_2_AlB_2_ weakened the interlayer bonding and enabled the etching of aluminum but requires toxic HF.^23^ When hydrothermal etching with 40% HF was applied to MoAlB,^24^ only part of the aluminum was successfully etched. MoAlB was partially etched in 2.5 M NaOH, producing MoBene along the edges, but the bulk of the sample remained MoAl_1–*x*_B.^25,26^ The problem with the synthesis of MBenes is that both the MBenes and aluminum are etched at roughly the same rate (as metals, they are both susceptible to oxidation), resulting in an absolute loss of selectivity.

We hypothesize that designing reaction conditions that favor an attack on aluminum oxyhydroxides will favor the formation of MBenes without resulting in sample degradation. We note that MAX and MAB phases, the crystallographic parent of MXenes and MBenes, are known for their facile formation of an aluminum oxide “scale” upon oxidation, which prevents further reaction.^27–29^ This was considered a boon in the past for the design of refractory barrier materials but now prevents effective etching. Thus, the ideal etchant would specifically target and remove the aluminum oxide/oxyhydroxide barrier (eq 1), thus exposing more aluminum to etching without needing the use of HF, a known oxide etchant. We are particularly inspired by the favorable reaction of aluminum oxyhydroxides with lithium ions (eq 2).^30^ In fact, simply exposing to lithium hydroxide will readily result in insertion between the edges of aluminum hydroxides at room temperature.^31^ We hypothesize that a mild hydrothermal treatment with lithium hydroxide will selectively target the aluminum oxyhydroxide scale of the MAB phase, exposing the underlying aluminum to further oxidation and repeated lithium ion attack (Figure 1).

1 $$MoAlB+H_{2}O+O_{2}\to MoB\left(OH\right)+AlOOH$$

2 $$2AlOOH+4H_{2}O+LiOH\to Li{Al}_{2}{\left(OH\right)}_{7}\cdot 2H_{2}O$$

**Figure 1:**
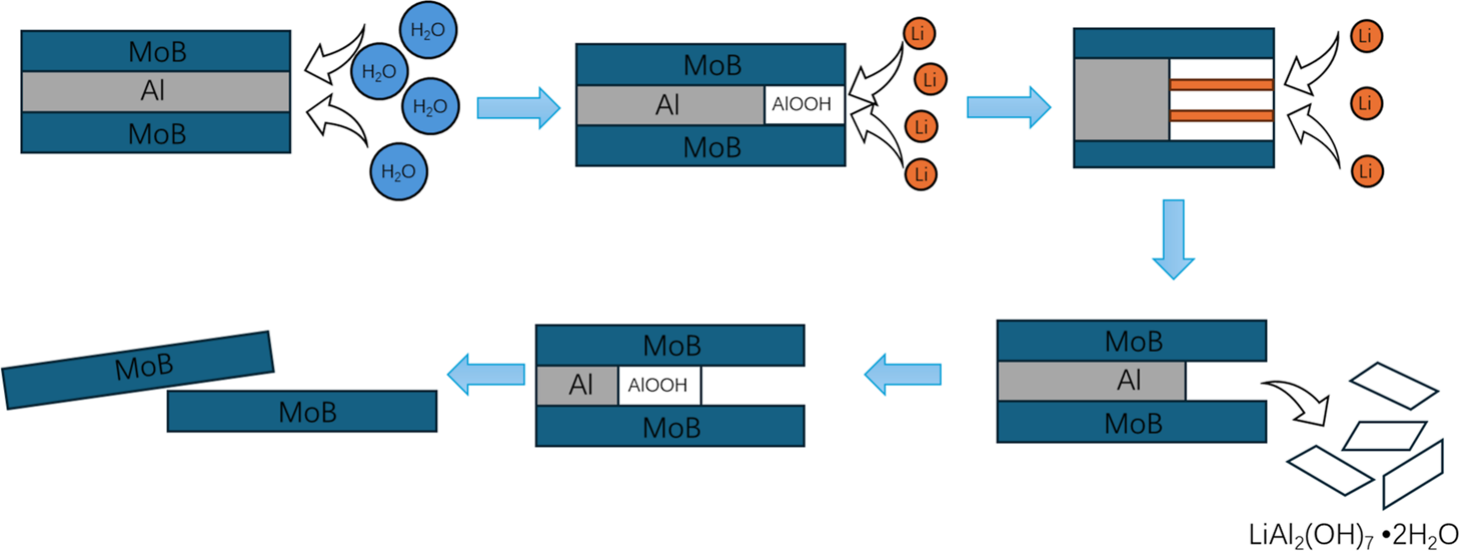
Diagram of LiOH facilitated water etching. Our mechanism of aluminum removal relies on LiOH that specifically targets the aluminum oxyhydroxide scale. Water as the etchant will react with the interlayer aluminum to form AlOOH and then Li^+^ will intercalate into AlOOH sheets to form LiAl_2_(OH)_7_·2H_2_O and draw out the aluminum.

MoBene is a single-layered MBene subunit of orthorhombic MoB (*Cmcm* no. 63), and etching MoAlB to MoBene would functionally yield nanolaminate MoB. In fact, the MoBene structural motif remains unchanged throughout our entire reaction scheme from bulk MoAlB to MoBene sheets (Figure 2). Bulk MoB has demonstrated unique applications in electrocatalysis^32^ and superhard metals,^33^ and a nanocrystalline modification would provide more access to the surface. More intriguingly, because of their structural similarity, the atoms do not need to be rearranged, thus avoiding the refractory nature of borides and avoiding sintering. This would enable a low-temperature route toward the synthesis of refractory borides.

**Figure 2:**
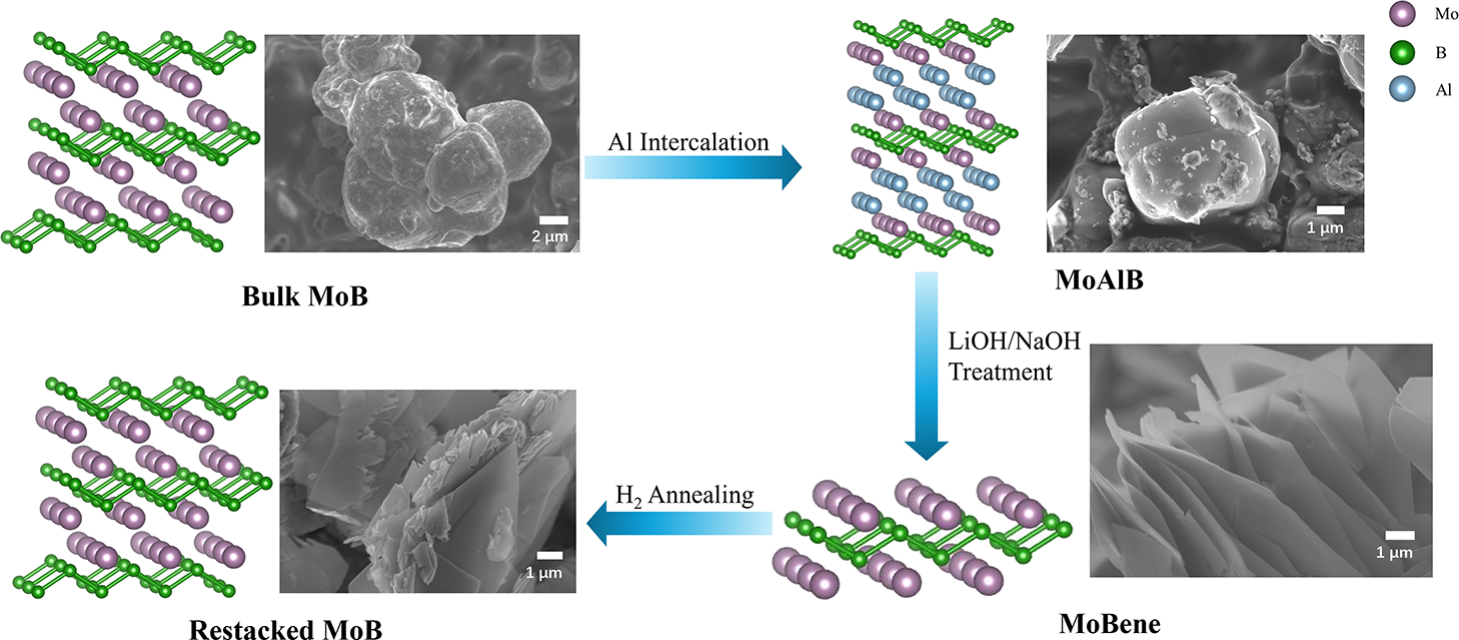
Illustration of transformation between MoB, MoAlB (MAB), and MoBene (MBene), and SEM images of each structure. Here, the MoBene subunit is maintained throughout all the topochemical processes, enabling low-temperature synthetic routes between structures.

In this paper, we develop a new low-cost, nontoxic, and environmentally friendly exfoliation approach by using dilute lithium hydroxide under hydrothermal conditions. The exfoliated MoBene sheets can be topotactically restacked to the parent MoB, confirming the high crystallinity of our MoBene sheets. The crystallographically evolution of our phases is shown in Figure 2.

## 2 Method

### 2.1 Synthesis of MoAlB

We follow the published method for the synthesis of MoAlB.^34^ MoB powder (99%, Alfa Aesar, USA) and aluminum powder (99%, Aldrich, USA) (molar ratio 1:1.3) were ground with a mortar and pestle until finely mixed. The mixed powders were then placed in an alumina boat and placed in a tube furnace under argon flow with a titanium getter placed upstream. The furnace was purged with argon for 6 h, then heated to 1000 °C with a heating rate of 5 °C/min, and held at that temperature for 15 h. The furnace was then cooled to room temperature at a rate of 5 °C/min, and the product was ground with a mortar and pestle to a fine black powder.

### 2.2 Hydrothermal Etching with LiOH

1 mmol of MAB precursor was mixed with 1 mL of 1 M LiOH (98%, Alfa Aesar, USA) and added into a 20 mL hydrothermal vessel in air. The vessel was heated in an oven to 200 °C, and the heating rate was 2 °C/min and held at the same temperature for 12 h. The product was first centrifuged at 4000 rpm for 4 min, and the liquid layer was decanted. The sample was then washed twice more with 30 mL of DI water, followed by washing with 15 mL of acetone three times, and dried in a vacuum oven for a minimum of 2 h.

### 2.3 Hydrothermal Removal of Lithium Aluminates to Yield MoBene

LiOH-etched MoAlB was heated with 10 mL of 5 M NaOH (97%, Fisher Chemical, USA) in a hydrothermal vessel to remove any Li–Al–O–H impurity. The vessel was heated in a convection oven to 130 °C at a heating rate of 2 °C/min and held at the same temperature for 12 h. The resulting MoBene products were washed with the same centrifuging procedure as that described above.

### 2.4 Restacking of MoB Sheets

Exfoliated MoBene powder, 1.8 wt % (NH_4_)_4_PtCl_2_·H_2_O powder (Thermo scientific, USA), and 1 mL of deionized water were mixed in a 2 mL centrifuge tube; the mixture was dried in a vacuum oven overnight. The platinum-impregnated powders were then loaded into a tube furnace under 5% H_2_ in argon, with a copper getter placed upstream to adsorb trace oxygen. The heating profile began with a 6 h purge, then it was raised to 120 °C for 6 h and heated to the desired temperature (*T* = 200, 250, 300, and 350 °C) for another 6 h. The sample was then analyzed using powder X-ray diffraction (pXRD) for restacking; then, the same sample was heated at the next temperature interval following the same heating profile.

### 2.5 Lead Adsorption

To get 1.5 mM PbCl_2_ solution, PbCl_2_ (99%, Thermo Scientific, USA) was mixed with DI water, the solution was heated on a hot plate at 60 °C overnight to dissolve PbCl_2_ completely, and the solution was cooled to room temperature before adsorption testing. MoBene was added to 45 mL of 1.5 mM PbCl_2_ solution in a ratio of 1 mg/mL, and the mixture was stirred on a stirring plate at 300 rpm at room temperature for 15 min. To separate the MoBene powder from the solution, the mixture was added to a 50 mL centrifuge tube and centrifuged at 4000 rpm for 5 min. The solution was saved for inductively coupled plasma atomic emission spectroscopy (ICP-AES) elemental analysis.

### 2.6 Instrumentation

The Teflon-lined hydrothermal vessels (Parr, 4749) were heated in a muffle oven (Heratherm, Thermo Fisher, OGH60). The synthesis of MoAlB and restacking of MoBene were performed in a tube furnace (Lindberg/Blue M Mini-Mite). The SEM images of exfoliated MoAlB were collected with a scanning electron microscope (Hitachi, Regulus 8100) under a 5 kV accelerating voltage and 10 μA of current. PXRD patterns were collected with a Rigaku SmartLab SE X-ray diffractometer using Cu Kα radiation (λ = 1.54 Å). Elemental analysis data was collected with an inductively coupled plasma atomic emission spectrometer (Shimadzu, ICPE-9820), and all the solid samples were first dissolved in aqua regia and then diluted 100-fold for ICP testing. The calibration curves for each tested element contain 6 data points: blank, 0.5, 5, 20, 35, and 50 ppm. Infrared imaging was collected with an FTIR Spectrophotometer (Shimadzu, IRTracer-100). Transmission electron microscopy (TEM) images were collected on an FEI Tecnai T12.

Standard laboratory safety procedures were followed. No uncommon hazards are noted.

## 3 Results and Discussion

Powder X-ray diffraction (pXRD) indicates the formation of phase pure MoAlB (Figure 3), which is the intercalated precursor to MoBene. Upon hydrothermal treatment with LiOH, the pXRD pattern (Figure S1) and EDS mapping (Figure S2) indicate the formation of LiAl_2_(OH)_7_·2H_2_O at the 1 h mark, confirming eqs 1 and 2 in our mechanism. MoAlB was partially etched to Mo_2_AlB_2_, and LiAl_2_(OH)_7_·2H_2_O was present as the byproduct of this etching procedure. The formation of MoBene sheets with strong exfoliated layer diffraction peaks is seen in the pXRD after the full 12 h reaction (Figure 3). Since LiAl_2_(OH)_7_·2H_2_O stability decreases at temperatures above 180 °C,^35^ it will decompose to amorphous AlOOH under our full 12 h at 200 °C reaction conditions (eq 3); therefore, it cannot be observed in the pXRD pattern; however, EDS mapping can find an aluminum–oxygen-rich impurity (Figure S3). It can also be proven by the ICP results in Table 1. The Li concentration in LiOH-treated MoAlB is lower than the detectable range, which indicates that the Li in LiAl_2_(OH)_7_·2H_2_O dissolved in the aqueous phase and was removed by centrifuging cycles.

3 $$Li{Al}_{2}{\left(OH\right)}_{7}\cdot 2H_{2}O\overset{above\;180{}^{\circ}C}{\to }2AlOOH\left(amorphous\right)+LiOH+4H_{2}O$$

**Figure 3:**
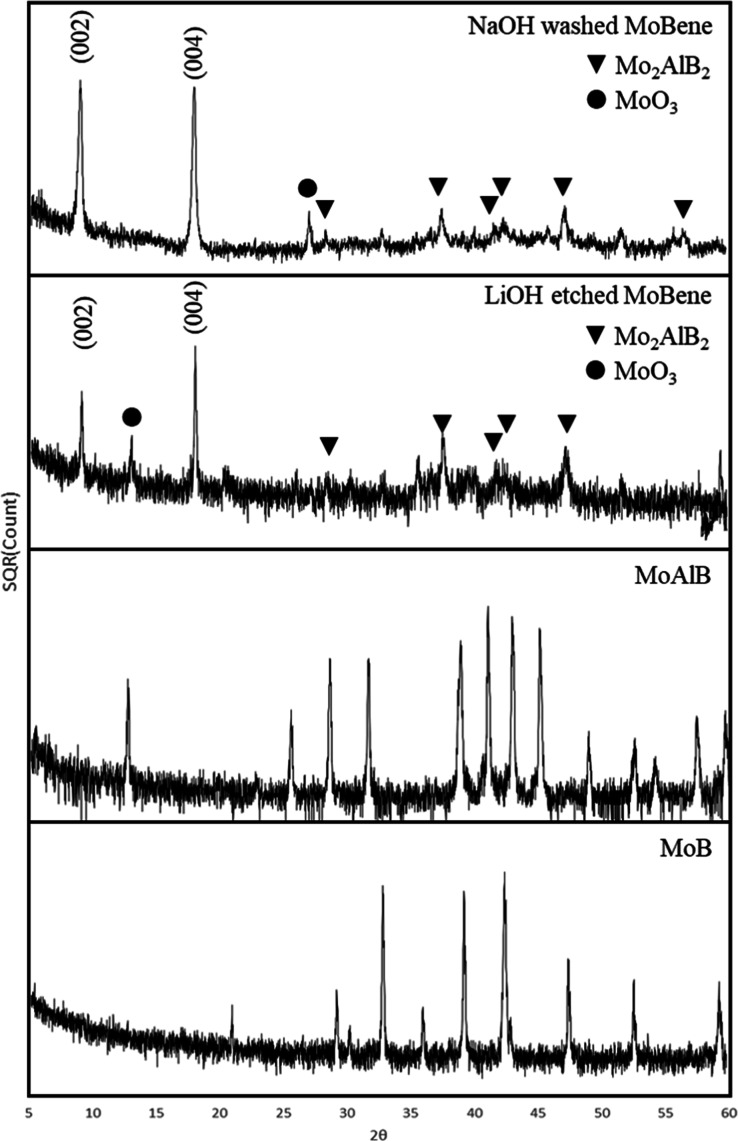
PXRD pattern of LiOH-etched MoB sheets, NaOH-cleaned MoB sheets, MoAlB precursor, and MoB precursor. MoBene interlayer peaks are indexed.

**Table 1:** Normalized ICP Results^a^

samples	concentration of elements (%)			
	Li	Al	B	Mo
LiOH- and NaOH-treated MoAlB	-	25.4	19.5	55.1
LiOH-treated MoAlB	-	46.7	18.5	34.8
MoAlB	-	29.9	34.2	35.9
MoB	-	-	50.3	49.7

a Concentrations of elements are normalized to molar percentage.

As expected from Bragg’s law of exfoliated layered materials, when there is high d-spacing, the pXRD pattern will show a peak at a low angle. The d-spacing of the peak at 8.9° corresponds to an interlayer spacing value of 9.9 Å. Based on the separation, the d-spacing must include twice the thickness of the surface functional group, which is hypothesized to be an –OH group based on alkaline etching methods. Since the thickness of a single layer of MoB sheet is ∼4.2 Å, as determined from the interlayer spacing found in the parent molybdenum monoboride, each of the –OH groups will have roughly half the length of the diameter of a water molecule, which is 2.75 Å. Assuming that a single layer of water is trapped between the sheets, the calculated interlayer d-spacing of 9.7 Å is very close to the measured value of 9.9 Å. As discussed above, unlike the room temperature HF-etched MXenes, our MoBene was surface-terminated with –OH groups. In addition, our reactions are in a water-based hydrothermal condition; the surface –OH groups will hydrogen bond with water and trap a thin bonded water interlayer. The two MoBene layers will be firmly bonded through the water layer, and the interlayer spacing will be held at 9.9 Å. Although the random stacking of the layered material will cause a variety of d-spacing, which will lead to a broad peak on the pXRD pattern, with the presence of strong interlayer hydrogen bonding, the layer registry is uniform at 9.9 Å for our sample, which will lead to narrow high-intensity peaks. The sharp peaks were found in MXene in a similar condition.^12^

Removal of the amorphous AlOOH via a 5 M NaOH treatment (eq 4) leaves behind the MoBene sheets with high reordering with the dominating (002) and the (004) peaks present.

4 $$AlOOH+NaOH+H_{2}O\to NaAl{\left(OH\right)}_{4}$$

To confirm the etching of aluminum from MoAlB and determine if any aluminum impurities remain after 5 M NaOH, inductively coupled plasma atomic emission spectroscopy (ICP-AES) was used to quantify the Mo, Al, and B content. As seen from Table 1, compared to MoB and MoAlB precursor, the majority of the aluminum was removed from the parent MAB phase and confirms the dissolution of interlayer aluminum; most of the remaining aluminum is likely amorphous aluminum oxide particles and thus would be absent in the pXRD pattern; the presence of the amorphous aluminum oxide impurity is confirmed with EDS mapping (Figure S3).

Lithium plays a key role in directly attacking the surface of aluminum oxyhydroxides. To confirm the need for lithium, MoAlB was hydrothermally treated under the same conditions with 1 mL of water without lithium hydroxide. The pXRD pattern of water-etched MoAlB before and after NaOH treatment is shown in Figure S4a,b, respectively. This matches the published pXRD pattern that claimed successful synthesis of Mo_2_AlB_2_.^36^ Here, MoAlB is only partially etched in water, as the byproduct, and as shown in Figure 1, aluminum oxyhydroxides serve as a barrier between the trapped interlayer aluminum and the oxidant water; therefore, aluminum cannot be completely removed. The presence of AlOOH is confirmed by pXRD (Figure S4a). The SEM image of water-etched MoAlB is shown in Figure S4d, and the AlOOH formed on the surface of MoAlB, after NaOH treatment (Figure S4e), the partially etched Mo_2_AlB_2_ is uncovered, while the AlOOH is removed.

Li^+^ facilitates aluminum oxyhydroxide removal and further etching of Al; furthermore, the low concentration of LiOH and relatively weak basicity prevent degradation of the MoBene sheets. Unfortunately, MoBene sheets are relatively fragile, ICP-AES indicates that 35% of the boron remains after all the etching steps, suggesting that there is an inevitable degradation that causes boron loss during the hydrothermal LiOH and NaOH treatment procedure, and the content of MoBene in the final product can be calculated to be around 38% by weight. The remaining Mo was oxidized to MoO_3_, which can be confirmed with the pXRD pattern in Figure 3. It also led to an excess of the Mo concentration in ICP results. A stack plot of the pXRD pattern (Figure S5) shows the presence of impurities in MoBene. It is worth noting that MoO_3_ only shows (020) and (040) peaks in MoBene before and after NaOH treatment, respectively. The Mo in MoO_3_ impurity is oxidized from the Mo in MoBene/MoAlB, which has layered structures. Additionally, MoO_3_ is known as a layered material as well;^37^ the MoO_3_ impurity will have a similar property as MoBene; it only shows the peaks that represent the base plane of the layers, which are (020) and (040) planes. Meanwhile, there is still Al present in our MoBene sample; most of the Al contents are from the amorphous aluminum oxide impurity as we discussed above, and the unetched Mo_2_AlB_2_ also provided parts of the Al contents. The presence of unetched Mo_2_AlB_2_ is confirmed with SEM (Figure S3) and pXRD (Figure 3).

The use of a hydrothermal treatment should result in the formation of surface functional groups. Here, we expect the formation of hydroxides that will trap water molecules between the sheets, which was confirmed with IR spectroscopy (Figure 4a). As seen in the IR, the broad peak at approximately 3400 cm^–1^ indicates the existence of surface hydroxide/trapped water. With the assistance of surface hydroxide groups, the MoBene sheets have a uniform dispersion in water, confirmed by the Tyndall effect (Figure 4b).

**Figure 4:**
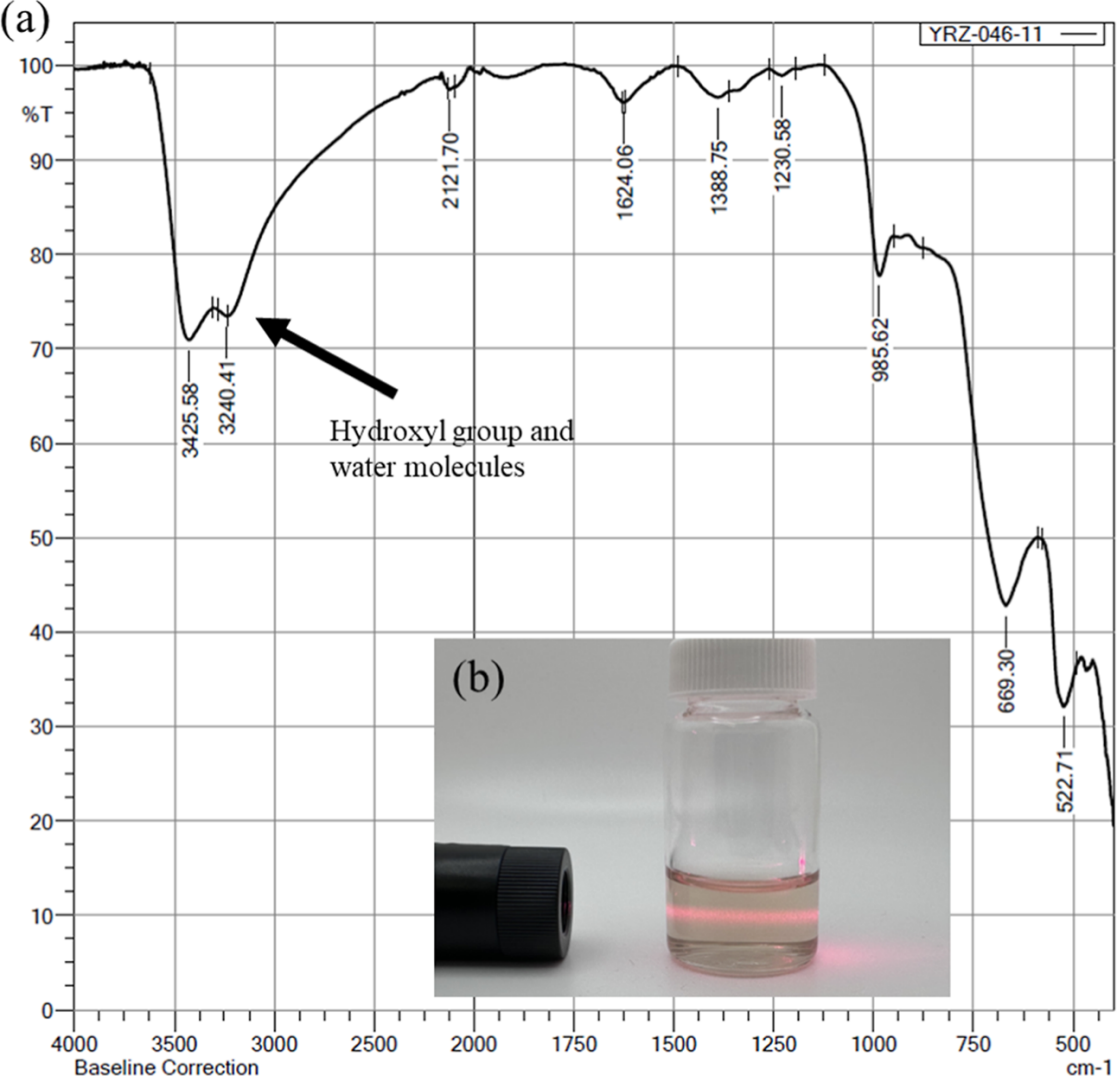
(a) FTIR image of NaOH-treated MoB sheets indicates that the surface of our MoBene is hydroxyl-terminated. (b) Tyndall effect of MoBene suspension after 24 h settlement.

Scanning electron microscopy (SEM) indicates the formation of exfoliated MoBene sheets (Figure 1), and the bulky MoAlB is exfoliated to MoBene sheets. Our as-prepared MoAlB presents itself as blobs, which is expected from our powder metallurgical approach. Upon etching, the morphology completely changes to sheets (Figure 1). Moreover, the high rigidity of the sheets as expected with a boride has resulted in very stiff sheets.^20^ Under the influence of the interlayer bonding between –OH groups and water, the sheets are distinct from the SEM images of MXenes in the literature; owing to the exceptionally stiff behavior of MoBene sheets, our sheets present themselves as rigid flakes. Transmission electron microscopy confirms the MoBene sheets are stacked upon each other (Figure S7).

To confirm the high crystallinity of our MoBene sheets, they were restacked back to their crystallographically related parent MoB. If there is any aluminum remaining between the sheets, it will either prevent restacking or form an impurity. Perhaps most intriguingly, the restacking of MoBene sheets to MoB would serve as a facile route toward the preparation of refractory MoB at low temperature. After all, MoBenes are the topotactically delaminated subunit of MoB, and it should be energetically accessible toward consolidation. To that end, we undertook a temperature study where the sample was heated, analyzed with pXRD to observe any convergence of layers/formation of MoB, and reheated at 50 °C increments.

To restack MoB sheets, the surface functional groups (hydroxyls and oxides) were removed with 5% hydrogen in argon/forming gas. Platinum is a hydrogenation catalyst that facilitates the formation of strongly reducing hydrogen radicals,^38^ which will increase the hydrogen reducing efficiency. The MoBene sheets were wet-impregnated with 1.8 wt % (NH_4_)_4_PtCl_2_·H_2_O powder and then heated in a tube furnace to 120 °C for 6 h to preremove adsorbed water and then heated to 200 °C, 250 °C, 300 °C, and 350 °C, respectively, for 6 h. In the pXRD pattern of the H_2_-treated sample (Figure 5), the (002) and (004) peaks shift to the right as the temperature increases, indicating the interlayer d-spacing is decreasing, while the peaks of MoB begin to reform at 350 °C. Note that the MoB peaks from the restacked sample are sharp and have low intensities; the sharpness of the peaks is formed through a phase transition under annealing, which removes the structural distortion.^39^ Because of the presence of impurities, only a few MoBene sheets that are not connected to impurities or interlayer Al can restack into small-scale MoB particles, which explains the low intensities of the peaks. The presence of restacked MoB is proved with the SEM image (Figure 1). Since the water and –OH groups are removed by H_2_ annealing, the weight loss of the sample represents the water and –OH content. In the pXRD pattern (Figure 5), after dehydration, the (002) and (004) peaks are broadened and shifted to the right. This indicates that the interlayer water is removed, the MoBene sheets are released from the H bonding between water and –OH groups, and the average d-spacing is smaller and spread in a larger range. A weight loss of 12.6% was observed after dehydration; since the weight content of MoBene in the final product is 38%, the water/MoBene molar ratio is 0.78.

**Figure 5:**
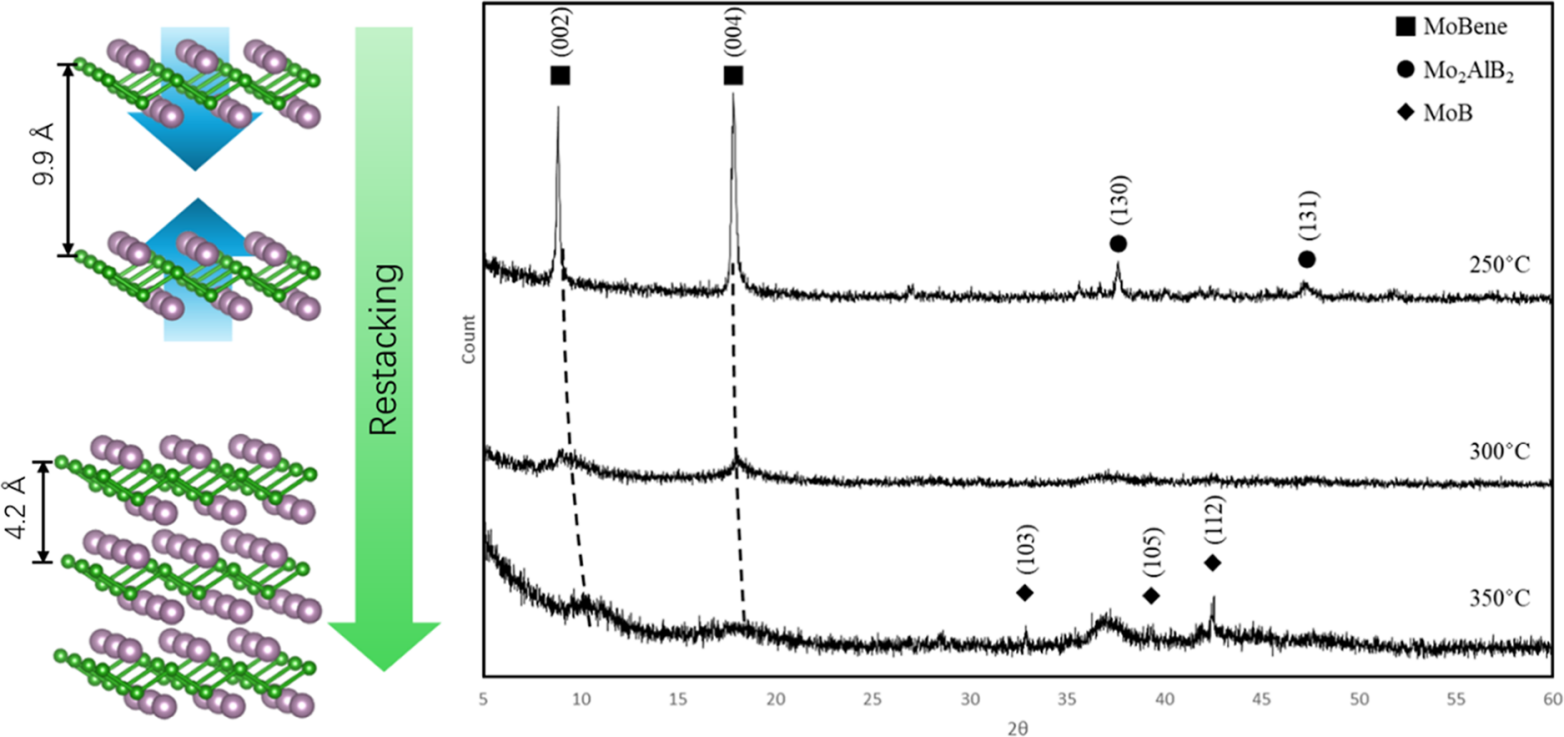
PXRD pattern of restacked MoB. The trend of the peak shifting is shown by the dashed lines. The low-intensity peaks in the 250 °C sample most likely belong to Mo_2_AlB_2_.

Given the clear changes at 300 and 350 °C, we hypothesize that restacking is a two-step procedure: first at 300 °C, interlayer water and surface hydroxyl groups are removed, and then at 350 °C, the sheets align and convert the free MoBene to MoB. Interestingly, this is the lowest temperature noted for the synthesis of borides. Typical boride synthesis requires temperatures greater than 1000 °C owing to the high melting point of boron.^40,41^ Because we are using the crystallographic subunit as a building block, where the metal and the boron have already been prearranged, we can synthesize high melting materials at relatively modest temperatures.

Finally, the hydroxyl-functionalized exfoliated MoBene sheets should readily chelate to potentially hazardous metals, and we show remarkable lead removal efficiency. To that end, we demonstrate that simply stirring MoBene in water is enough to remove heavy metals, such as lead. As determined by ICP-AES, the adsorption capacity of 318 mg/g is obtained by stirring MoBene powder in 1.5 mM PbCl_2_ in a ratio of 1 mg/mL for 15 min, and the adsorption rate is calculated by eq 5.

5 $$Q=\frac{\left(C_{i}-C_{f}\right)VM}{m}$$

where *Q* is the adsorption rate, *c*_*i*_ is the initial concentration, *c*_*f*_ is the final concentration, *V* is the volume, *M* is the molar mass of lead, and *m* is the mass of MoBene used. As a comparison, the lead adsorption capacity of MXene Ti_3_C_2_F_*x*_ is only 23 mg/g, as the fluorine termination is significantly less binding. To understand the morphology, MoBene after Pb adsorption is studied with SEM and EDS (Figure S8). The SEM image indicates that the MoBene sheets have an increased surface roughness after adsorption; the EDS mapping confirmed that there is Pb aggregate on the surface of the MoBene sheets. Since the MoBene sheets are not completely covered with Pb particles, we predict our MoBene material could have better performance in Pb adsorption under different conditions.

## 4 Conclusion

A low cost and environmentally friendly exfoliating method was demonstrated for MoAlB by simply heating with dilute lithium hydroxide. Using this method, complete exfoliation of the MoB sheets is achievable. As the crystallographic subunit of MoB, MoBenes readily restack at low temperatures, enabling the synthesis of high-temperature refractory materials. The exfoliated MoB sheets are surface-functionalized with hydroxyl groups. The product shows a remarkable lead adsorption rate of 318 mg/g, which opens a broad future for MoBenes in heavy metal adsorption.

## Supplementary Material



## Notes

Additional pXRD patterns of the products from different stages, EDS mapping of MoBene- and Pb-adsorbed MoBene, SEM images of water-etched MoAlB, picture of MoBene suspension in water, and TEM image of MoBene (PDF)
