# Supplementary material for: A Mild Alkaline Hydrothermal Etchant for MBene Exfoliation
Source: Inorg Chem. 2025 Sep 11;64(37):18696–703. doi: 10.1021/acs.inorgchem.5c01969 (PMC12458703; doi:10.1021/acs.inorgchem.5c01969)
Supplement: Supplementary file 1 [file ic5c01969_si_001.pdf]

Supporting Information

A Mild Alkaline Hydrothermal Etchant for MBene Exfoliation

Yiren Zhang[1], Abraham A. Rosenberg[1], Joseph T. Doane[1], William Rice[1], Alma Kolakji[1]; Michael T. Yeung[1*]

[1] Department of Chemistry; University at Albany SUNY; Albany, New York, 12222

[*] Corresponding authors: mtyeung@albany.edu

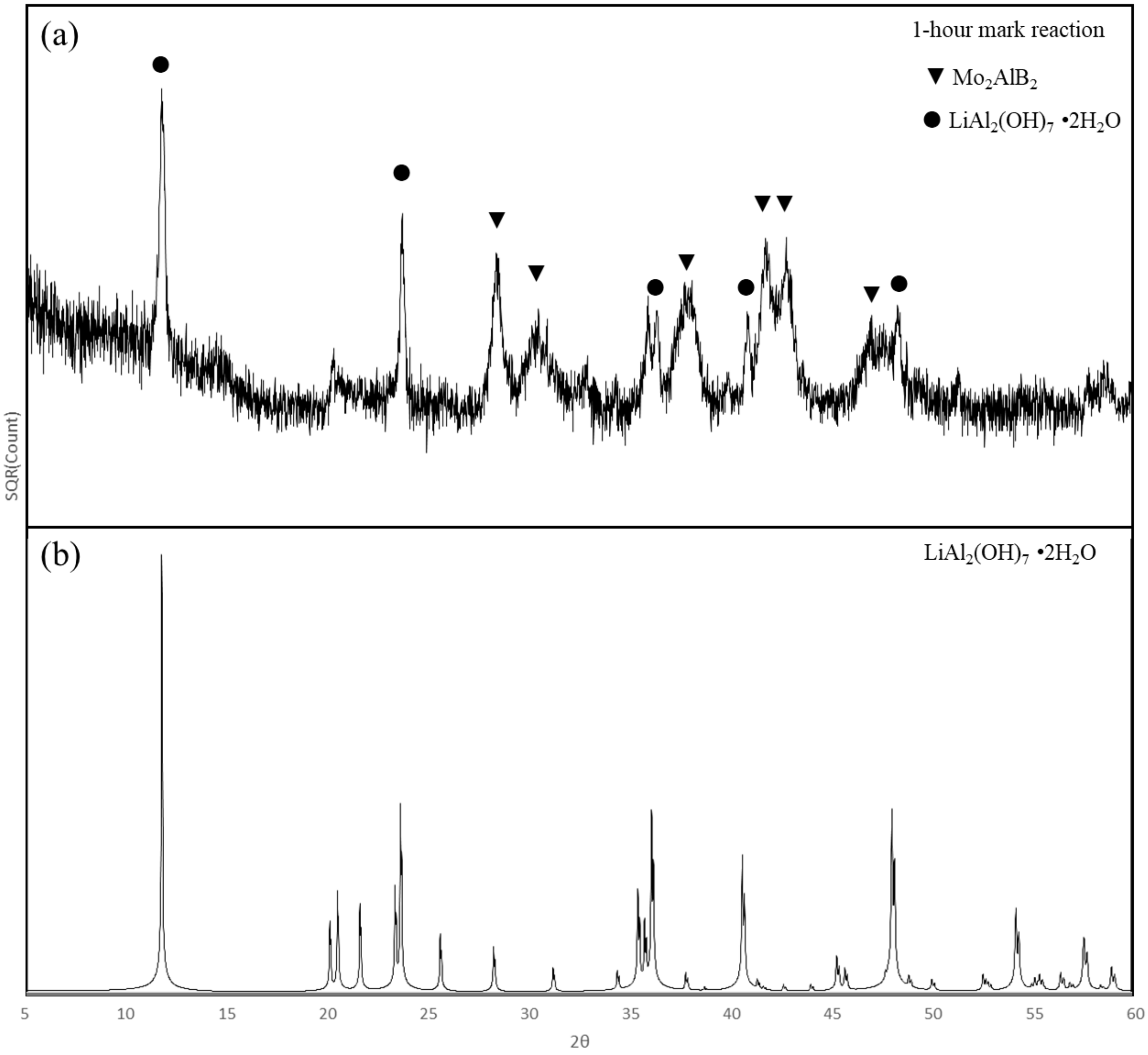

Figure S1. PXRD pattern of (a) MoAlB etched in LiOH for 1 hour, (b) $LiAl_2(OH)_7{\bullet}2H_2O$[1].

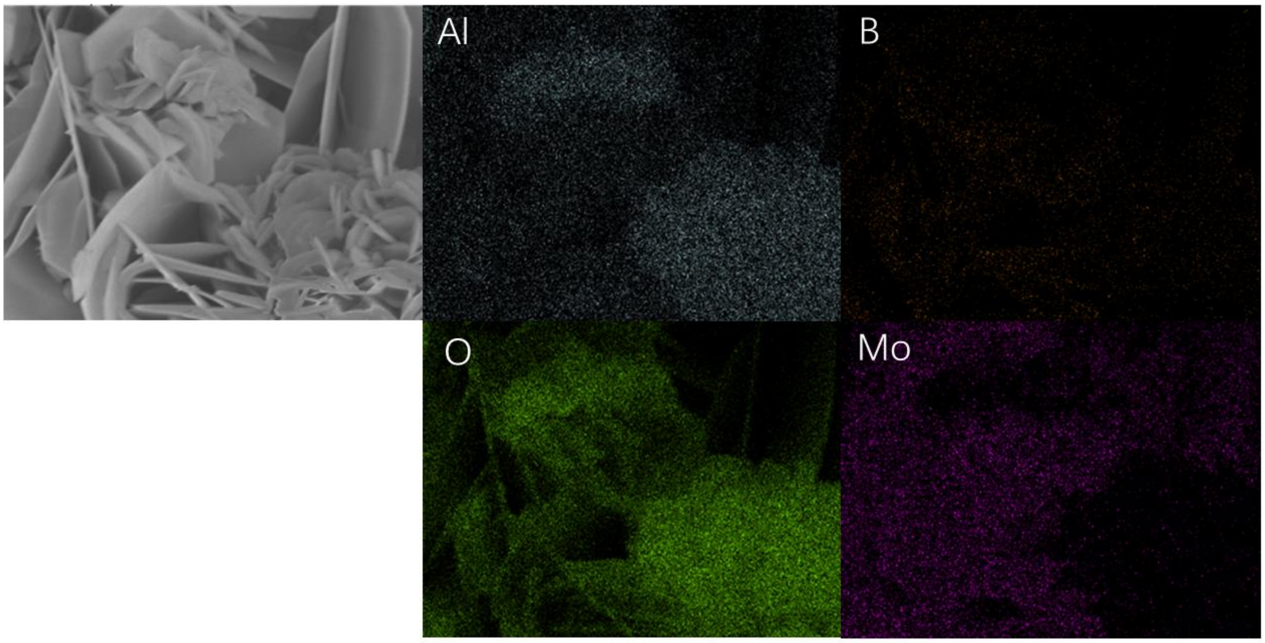

Figure S2. Compositional maps of Al, B, O, and Mo in LiOH treated MoAlB with aluminum oxide impurity.

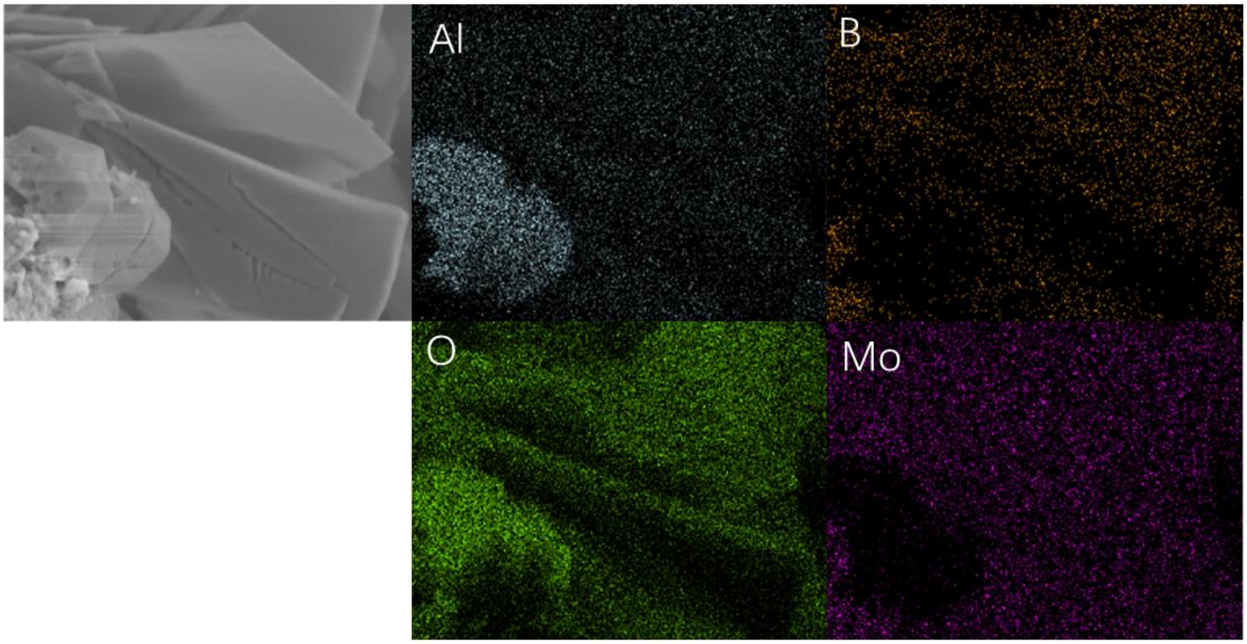

Figure S3. Compositional maps of Al, B, O, and Mo in exfoliated MoB sheets with aluminum oxide impurity.

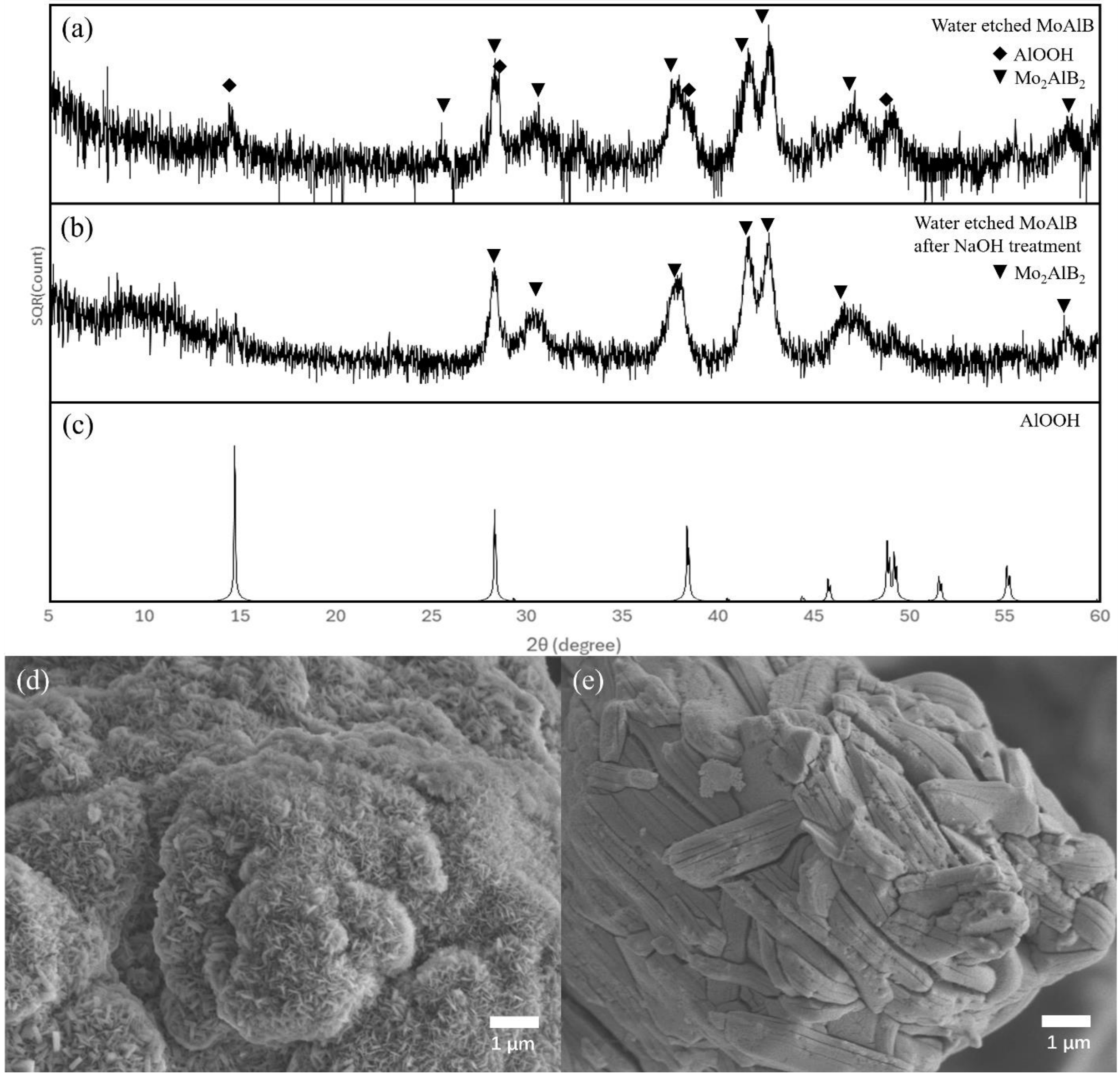

Figure S4. PXRD pattern and SEM images of water etching products. (a) PXRD pattern of water etched MoAlB. (b) PXRD pattern of water etched MoAlB after NaOH treatment. (c) PXRD pattern of AlOOH[2]. (d) SEM image of the products of water etching before NaOH precudure. (e) SEM image of the products of water etching after NaOH treatment.

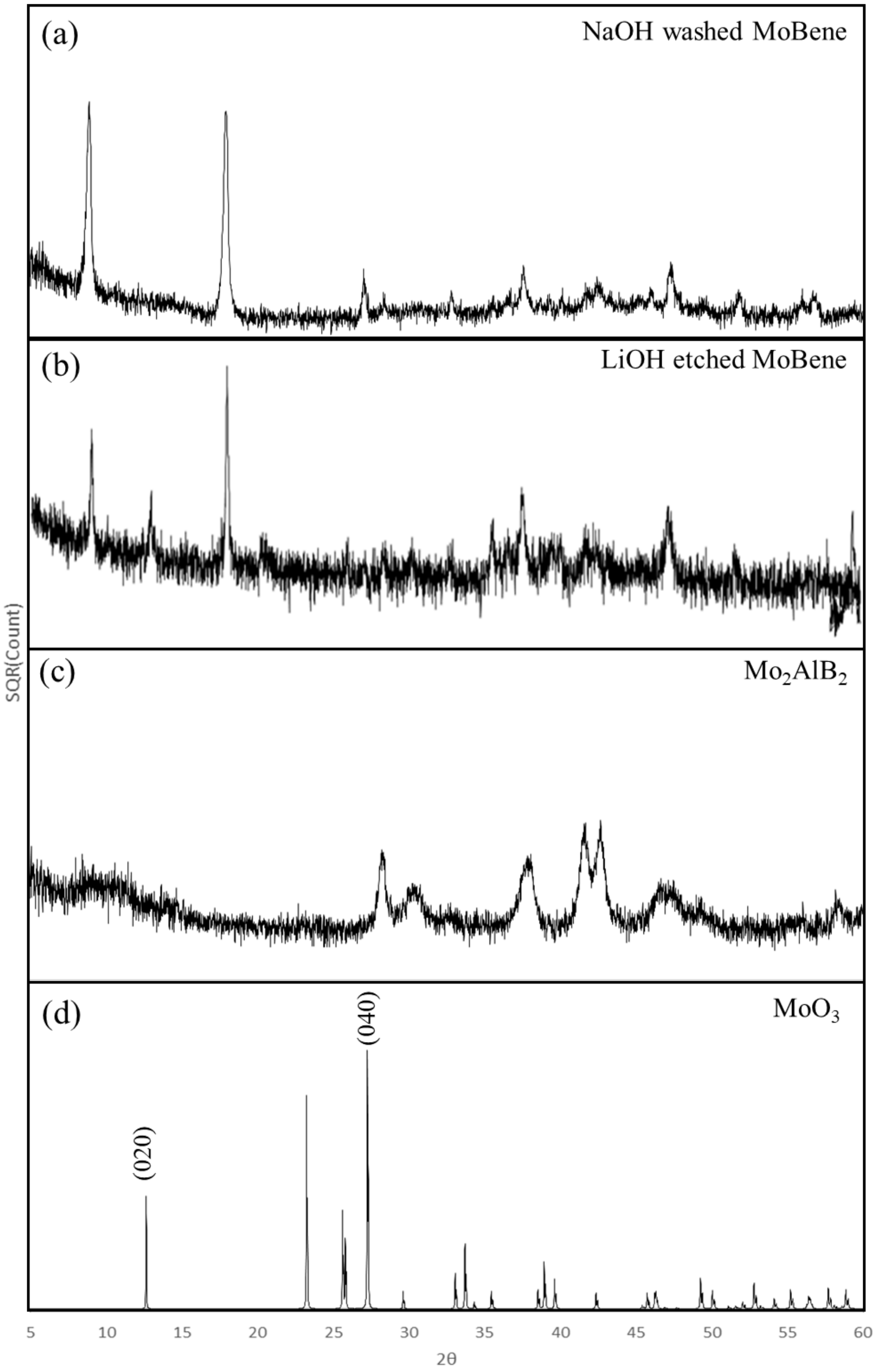

Figure S5. PXRD patterns of (a) NaOH washed MoBene, (b) LiOH etched MoBene, and impurities (c)Mo_2AlB_2, (d)MoO_3[3].

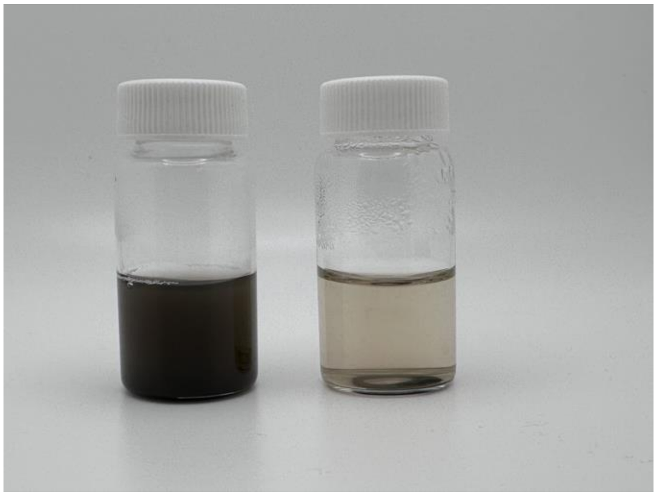

Figure S6. MoBene dispersion in water after 1h sonication (left) and after 24h settlement (right).

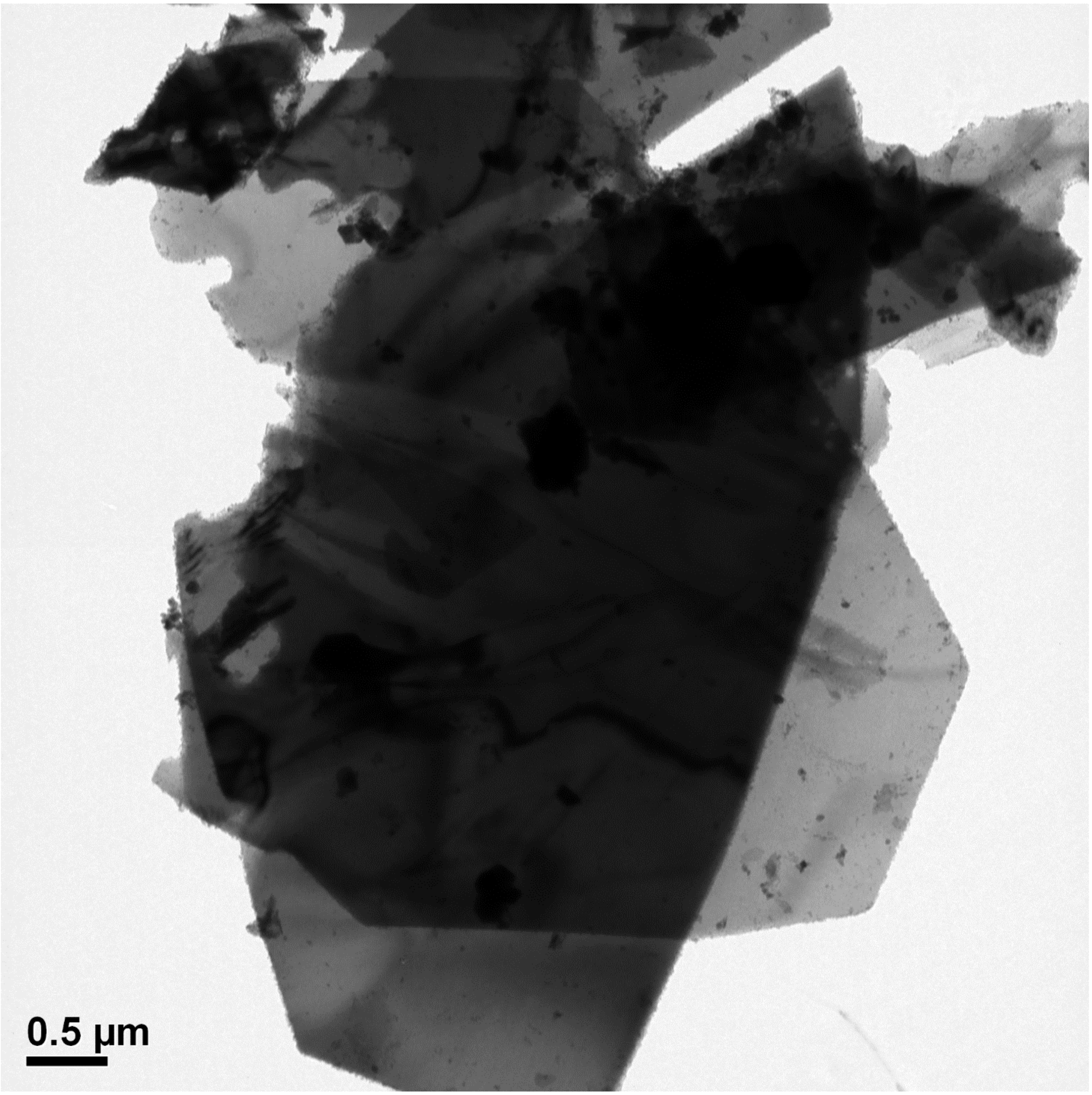

Figure S7. Transmission Electron Microscopy of MoBene, indicating a stacked layered structure.

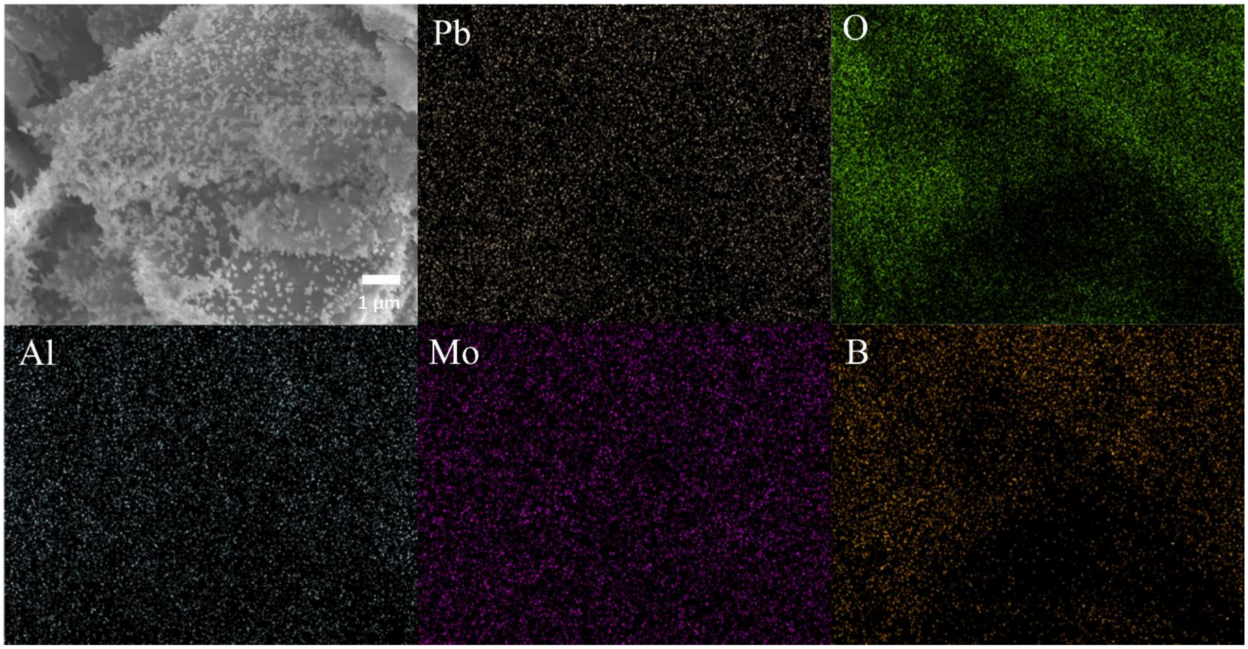

Figure S8. SEM image and EDS mapping of MoBene after Pb adsorption.

References

(1) Thiel, J. P.; Chiang, C. K.; Poeppelmeier, K. R. Structure of Lithium Aluminum Hydroxide Dihydrate (LiAl2(OH)7.2H2O). *Chem. Mater.* **1993**, *5* (3), 297–304. https://doi.org/10.1021/cm00027a011.

(2) Hill, R. J. Hydrogen Atoms in Boehmite: A Single Crystal X-Ray Diffraction and Molecular Orbital Study. *Clays and Clay Minerals* **1981**, *29* (6), 435–445. https://doi.org/10.1346/CCMN.1981.0290604.

(3) Wooster, N. The crystal structure of Molybdenum Trioxide, MoO3. *Zeitschrift für Kristallographie - Crystalline Materials* **1931**, *80* (1–6), 504–512. https://doi.org/10.1524/zkri.1931.80.1.504.
